# Evaluating the Preventive and Therapeutic Roles of Active Irrigation Systems in Root Canal Treatment: A Narrative Review and Critical Appraisal of Theory and Methodology

**DOI:** 10.3390/dj13010009

**Published:** 2024-12-26

**Authors:** Ignacio Barbero-Navarro, Iuliana Sofian-Pauliuc, Maria Esther Irigoyen-Camacho, Marco Antonio Zepeda-Zepeda, David Ribas-Perez, Antonio Luis Castaño-Seiquer

**Affiliations:** 1 Dental School, University of Seville, 41009 Seville, Spain; ibarbero2@us.es (I.B.-N.); iulsofpau@alum.us.es (I.S.-P.); acastano@us.es (A.L.C.-S.); 2 Health Care Department, Autonomous Metropolitan University-Xochimilco, Mexico City 04960, Mexico; meirigo@correo.xoc.uam.mx (M.E.I.-C.); mzepeda@correo.xoc.uam.mx (M.A.Z.-Z.)

**Keywords:** active irrigation systems, chemical debridement, root canal system, therapeutic efficacy, prevention, ultrasonic irrigation, sonic irrigation

## Abstract

Endodontic therapy aims at preventing or curing apical periodontitis. To conduct this, the cleaning and shaping of the canals are essential. By using an irrigant, such as sodium hypochlorite (NaOCl), practitioners attempt to wash out debris, dissolve organic and inorganic tissue, lubricate the canals, prevent smear layer formation, and disrupt biofilms. Different methods have been developed to optimise the effectiveness of irrigants, including manual or passive techniques (the conventional method used worldwide) and active techniques (the irrigant is activated by certain devices to improve its flow in the root canal system). Some techniques included in the active category are ultrasonic and sonic methods, apical negative pressure irrigation, and multisonic activation. These active techniques appear to have higher effectiveness when compared to the conventional syringe method during the root canal treatment procedure. However, it is unclear whether they also have a higher influence on the treatment outcome and healing of apical periodontitis. There is a consensus on the need for endodontic studies with standardized protocols and methods to reach a standardized clinical protocol when using active irrigation.

## 1. Introduction

Root canal therapy is the dominant strategy used to remove the intracanal infection caused by bacteria in a tooth and address apical periodontitis. The disinfection and decontamination of the root canal system are key for debriding organic and inorganic tissues; thus, chemo-mechanical preparation is crucial for treatment success. This treatment not only requires mechanical preparation but is also dependent on the chemical and biological effectiveness of the irrigant [1,2,3,4,5].

The irrigants most used are sodium hypochlorite (NaOCl) and ethylenediamine tetra-acetic acid (EDTA), with different methods used to deliver them in the root canal [2,4,6]. NaOCl is the choice in endodontics due to its efficacy and properties. Chlorhexidine (CHX) is the third type of irrigant used as an alternative to NaOCl, although its effectiveness is still debated [7].

Therefore, it is advised to exchange irrigants more frequently and in larger quantities to increase their efficacy along with employing smaller irrigating needles and inserting them deeper into the canal [8].

### 1.1. NaOCl, EDTA

Clinicians attempt to obtain the best cleaning effectiveness possible using NaOCl and EDTA. These irrigants have flaws, yet they are the closest to the ideal type of irrigant. They achieve the best results by lubricating the canal, eliminating organic and inorganic material, flushing out debris, and having an antibacterial effect [4].

Several variables must be considered to reduce the amount of organic and inorganic material since they could have an impact on irrigant penetration:

− The irregularities of the root canal anatomy.
− The delivery mode, volume, and physical and chemical properties of the irrigant and the presence of gas bubbles [5].

These factors make it necessary for NaOCl to reach the apex in sufficient concentration and quantity, where microorganisms are more likely to survive as it is filled with irregularities (delta, fins, ramifications, and isthmuses) [5,9].

### 1.2. Types of Irrigation Systems

Two categories of irrigation systems can be distinguished to deliver the irrigant in the root canal system:

− Manual or static technique: The irrigant is delivered passively through a syringe with a side-vented needle. This is called positive pressure irrigation (PPI). This method is also known as conventional needle irrigation (CNI).
− Active or machine-assisted techniques: The irrigant is activated by certain devices to improve its flow in the root canal system. Some types included in this category are ultrasonic and sonic methods, apical negative pressure irrigation, and multisonic activation. This group is also known as IATs (irrigant activation techniques) [5,10,11,12,13,14,15].

#### 1.2.1. Syringe or Conventional Irrigation

Manual irrigation is a positive pressure system. This includes the use of a syringe (using a small gauge needle: 30 gauge or less) and a specific method of delivering the irrigant. The needle tip needs to be placed 1–2 mm away from the working length in the root canal, meaning that the flushing action and cleaning of the apical portion and isthmuses are limited. By applying this approach, the risk of apical extrusion and the creation of complications are avoided [3,9,13,16,17].

In CNI, the irrigant is delivered by pushing the syringe needle up and down in the canal like a piston (it is advised to perform this action slowly to control manual pressure). The physical characteristics of the needle, where it is inserted into the canal, and its anatomy all have a significant role in how well this method can flush the canal [18].

Mozo et al. [19] explained that if syringe irrigation is used, it is recommended to not bend the needle or place it too close to the WL and to be gentle in delivering the irrigant so it does not penetrate beyond the periapex.

Research has demonstrated that CNI does not effectively remove debris and smear layers from root canal irregularities. Because of this, clinicians are switching more and more to machine-assisted irrigation activation methods, although CNI is the most used technique worldwide. IATs appear to remove more debris and intracanal smear layers when compared to CNI [14,18].

There are several methods for the activation of the irrigant, such as manual dynamic activation (MDA), which means repeatedly inserting the gutta-percha (GP) cone into the working length using 2–3 mm longitudinal up-and-down strokes to create hydrodynamic pressure that moves the irrigant, file agitation, and simply hand agitation. The GP cone has to be tapered, adapting it to the root canal wall [3,9,13,14,16,17].

#### 1.2.2. Active Irrigation Systems

Irrigant activation has increased in popularity and development, due to the improvement in removing debris from the apical and non-instrumented areas of the root canal system by using a combination of suction, vacuum, oscillation, or pressure. Nevertheless, there are no standardized protocols on the use of these devices in clinical practice and their application will only depend on the clinician who is using them.

Using lasers or sonic or ultrasonic equipment to change the irrigant’s flow is a common method of activating the final rinse of the irrigant. Encouraging the irrigant to move creates shear forces that improve the removal of debris and smear layer from the canal walls and expose the dentine tubule entrance. Once uncovered, the irrigants and medications could penetrate more easily into the dentine [18].

##### Passive Ultrasonic Activation (PUI)

PUI uses oscillating non-cutting files at ultrasonic frequencies (25–30 kHz) and creates waves that induce acoustic streaming (circular and rapid movement of the irrigant around the vibrating file) and cavitation (creation and distortion of bubbles) in the irrigant (the file is placed in the canal filled with irrigant). This technique allows the irrigant to be activated without instrumenting the root canal system and to move dynamically. The irrigant can be flushed continuously or intermittently (having the same effectiveness in removing dentine debris), appearing to be more effective than CNI [16,17,18,20,21].

The ultrasound waves intensify the penetration of the irrigant in complex anatomic areas, improving the cleaning ability [16,22,23].

Nevertheless, in clinical conditions, it is nearly impossible to prevent file-to-wall contact with ultrasonic devices, which can result in small dentine loss. The power setting in ultrasonic irrigant activation affects the amplitude of the file oscillation, which has a significant impact on the streaming and cleaning efficacy of the irrigant. It has not yet been determined if varying power settings affect acoustic irrigant activation effectiveness in a comparable way [24].

##### Proultra Piezoflow^®^

This device was designed by Dentsply Sirona (Charlotte, NC, USA). It can be attached to a conventional syringe and to an ultrasonic handpiece at the same time. The irrigation here is delivered continuously (known as CUI, continuous ultrasonic irrigation).

##### Irrisafe^®^

Irrisafe (Satelec Acteon Group, Merignac, Paris, France) is an ultrasonic device that activates the irrigant solution through acoustic streaming and micro cavitation; this technique is PUI, and it allows for the delivery of irrigants up to the working length (WL) of the root canal, unlike a conventional endodontic needle [9,25].

#### 1.2.3. Sonic Activation (Si)

This agitation technique uses a low sonic frequency (1–10 kHz) to generate streaming of the irrigant, a hydrodynamic phenomenon. The file is a smooth flexible polymer and has a transverse oscillation. The file can come in multiple sizes and tapers without the risk of dentine removal [11,16,17,24].

##### EndoActivator^®^

EndoActivator (Dentsply, Tulsa Dental Specialties, Tulsa, OK, USA) is a sonic device that comes with three different polymer sizes and a handpiece that has three different speeds (high, medium or low). The EndoActivator is suggested to be used after root canal shaping and flushing with manual irrigation as it promotes greater diffusion into the canal irregularities. Previous research shows greater removal of the smear layer, exposing more dentinal tubules when compared to CNI, and is relatively safe as there is a low chance of creating additional debris if it touches the canal walls [9,17,18,25,26].

##### Vibringe^®^

The Vibringe (Vibringe BV, Amsterdam, The Netherlands) system uses sonic vibration added to the traditional syringe to deliver the irrigant. Compared to conventional irrigation, Vibringe removes more debris in the apical region because of its higher fluid velocity due to the oscillation amplitude of the tip, which is higher at the end of the file than at the connected end. Its cordless handpiece fits into a unique, disposable 10 mL Luer-Lock syringe that can be used with any kind of irrigation needle [10,16,17].

#### 1.2.4. Pressure Alternation Devices

##### Endovac^®^

An apical negative pressure irrigation device called Endovac (KerrEndo, Orange County, CA, USA) uses a microcannula at the WL to remove the irrigant solution.

As a result, there is no risk of extrusion past the apical foramen and the vapour lock effect. It takes a significant amount of apical preparation (up to size 40) to insert the cannula into the canal, which could be challenging to accomplish in curved canals [9,16,25].

##### RinsEndo^®^

The RinsEndo^®^ device (Dürr Dental GmbH, Bietigheim-Bissingen, Germany) is also a negative pressure irrigation. It has an ultra-thin flexible cannula, which is placed in the apical third of the root canal and works via hydrodynamic activation of the irrigant [16].

#### 1.2.5. Continuous Irrigation During Instrumentation

##### Self-Adjusting File^®^

A hollow, flexible file called the Self-Adjusting File (ReDent Nova, Ra’anana, Israel) conforms to the root canal in three dimensions. There is minimal dentine removal and a vertical vibration motion (like scrubbing). This is delivered during the cleaning and shaping process through the filing system (continuous irrigation). The irrigants do not effectively reach the apical part because this system does not have control of the apical enlargement. It was also suggested that it is not ideal for oval canals as they can jeopardise the outcome of the treatment due to possible metal particles released [9,16,25].

#### 1.2.6. Multisonic Activation 

This type of activation improves the irrigant flow and chemical debridement in the root canal system by imploding the bubbles created by acoustic waves in different frequencies.

##### GentleWave^®^

GentleWave (Sonendo, Laguna Hills, CA, USA) is a device using this method. The shaping of the canals is minimal (apical size of 15–25), and the irrigation is delivered through a tip into the pulp chamber connected to a handpiece. The excess is also withdrawn by a vented suction attached to the handpiece.

Acoustic energy and cavitation are used to reach those complex areas and microscopic spaces. At the same time, gases are eliminated because of the optimised fluid. The whole process takes between five and eight minutes [16,27].

#### 1.2.7. Rotary File Agitation

##### XP-Endo Finisher^®^

The XP-Endo Finisher (XPF, FKG Dentaire, Le Crêt-du-Locle, Switzerland) consists of a flexible rotary Ni-Ti file that broadens into a sickle shape at body temperature but is linear at room temperature. The file is size 25 with a zero-taper. This enables the file to better adapt to the anatomy and lower the damage to the root canal [17].

#### 1.2.8. Laser Activation

##### Laser-Activated Irrigation (LAI)

“An end or radial firing tip” is used to deliver laser energy inside the root canal. It is positioned in the apical third of the canal and has the shape of a cone with a 60° angle, allowing the laser light to be delivered towards the apex and expand to cover the root canal more evenly [16,28].

By using intense absorption energy to cause optical breakdown, LAI produces transient cavitation in the liquid. The radiation transmitted in the solution promotes the generation, expansion, and burst of the bubbles. The conclusion was that LAI (SWEEPS and PIPS) was more effective in removing hard tissue debris than ultrasonic activation if activated for 20 s [16,17,29].

##### Photon-Induced Photoacoustic Streaming (PIPS)

The tip is modified and placed in the pulp chamber, submerged in the irrigant repeats. It is used three to four times for 20 to 30 s each time using “short low-energy pulses” (50 μs, 20 mJ) at a rate of 15 Hz.

Both types of lasers use pulse mode and are highly absorbed in water. The irrigant is activated by using “Erbium: Cr and YAG” [16,17].

The Er: YAG (erbium-doped yttrium aluminium garnet) and Er, Cr: YSGG (erbium-doped chromium–yttrium–scandium–gallium garnet) lasers have conically shaped, radial firing tips that irradiate the root canals in three dimensions, leading to more widespread bacterial death [21].

##### Shock Wave-Enhanced Emission Photoacoustic Streaming (SWEEPS)

This is LAI’s most recent advancement. It employs two pulses to produce a stronger shock wave. A primary and secondary cavitation effect is created by delivering the laser pulses in pairs, with a gap of around 600 μs between each pulse. It is thought that more root canal regions are touched by secondary cavitation. However, more research is needed on its cleaning effect [17].

#### 1.2.9. Gas Bubbles or Vapour Lock

A liquid’s penetration into a cavity depends on its capillary forces, contact angle [angle formed by a drop of liquid at its three phases, where liquid, gas, and solid intersect, assessing the wettability of the surface], viscosity, and size. Once it penetrates, it can flood the cavity entirely or entrap the gas or vapour. This will be conditioned by the type of cavity [closed or open]. Vapour lock happens when air is mainly entrapped in the apical third of the root canal and influences how the irrigation and debridement work [5].

In a closed cavity, such as the tooth, the movement of gas bubbles and the dynamics of fluids are affected by the degree of inclination of this cavity, thus producing a vapour lock effect, which will impede the irrigant from reaching the apical third. The gas bubbles can be removed by using a patency file and ultrasonic activation of the irrigant [5].

− The vapor lock effect can be avoided by enlarging the root canal and having the needle close to the working length as this effect occurs more when using conventional needle irrigation. However, the conventional needle should not be closer than 1–2 mm to the working length due to the risk of apical extrusion of the irrigant [7].

#### 1.2.10. Acoustic Streaming and Cavitation

“The intense circular fluid movement or flow patterns around files” is what is known as “acoustic streaming”. When the non-cutting files are engaged with passive ultrasonic energy, the irrigant increases the debridement in the canal due to “hydrodynamic shear stress” [22,30].

The problem with these files is that they bend or have a restricted vibratory motion in curved canals, reducing the cleaning efficiency. This would be resolved by having a smaller file that vibrates freely in the canal, at a high-power setting [6,31].

These energy or acoustic waves dissipate through the irrigant, leading to cavitation and microstreaming (the energy released when the bubbles collapse helps the debris reach the coronal part and be removed). This cavitation can be either transient cavitation (violent collapse of the bubble) or stable cavitation, which is a gentle oscillation [7,32].

##### The Smear Layer

This layer is a covering of organic and inorganic material produced during root canal treatment (by-products of instrumentation). It is composed of dentine, pulpal remnants, odontoblasts, and microorganisms on the canal walls. If it is not removed during the process, the root filling will be compromised as the sealers will not have the capacity to penetrate the dentinal tubules found on the canal walls [6].

The purpose of researching active irrigation and its influence on chemical debridement is to compile a wide variety of studies, analyse them, and filter and bring up-to-date available literature. This could help condense and reduce the overload of studies on such an important topic in endodontics.

In the selected studies reviewed, relevant data were identified: study design, type of irrigation system evaluated, outcome measured, and other methodological areas. Key themes such as the preventative and therapeutic roles of active irrigation were emphasised.

By retrieving and assessing what is already published, this narrative review provides a comprehensive understanding of the topic to help provide new insight into the advancing field or future direction and perspective in the use of active irrigation while performing root canal therapy. Thus, new possible paths for development in this field or a more definitive guideline in active irrigation sound interesting.

## 2. Materials and Methods

A comprehensive literature search was conducted to identify relevant studies on the topic. Several databases were consulted for selecting articles, such as PubMed, Cochrane, ScienceDirect, and Central, and limited to the years between 2000 and 2024 in the English language.

Some of the keywords included “root canal treatment”, “chemical debridement”, “active irrigation systems”, “therapeutic efficacy”, “prevention”, “ultrasonic irrigation”, or “sonic irrigation”.

### 2.1. Search Strategy and Databases

Terms Used:

− PubMed: “active” and “irrigation” and “system”, “chemical debridement” AND “root canal system” AND “therapeutic efficacy” AND “prevention” AND “ultrasonic irrigation” or “sonic irrigation”.

This search resulted in 81 articles (between 2003 and 2024).

− Scopus: “chemical debridement” AND “root canal system” AND “ultrasonic irrigation” OR “sonic irrigation”.

This database produced 104 results (between 2002 and 2023).

− Other databases such as Wiley, ScienceDirect or Cochrane: “chemical debridement” AND “root canal system” AND “ultrasonic irrigation” OR “sonic irrigation”.

84 articles appeared in the Wiley database (2002–2024).

### 2.2. Study Selection

First, duplicate studies were eliminated and noted. The remaining studies underwent second-stage searching, which involved reading the entire text, and 70 articles were initially selected for this review to give and present a wider and more comprehensive view of the topic.

To be able to synthesise and find the most relevant data, 16 articles were included in the final discussion of this review and had to meet one or several of the following inclusion criteria:

− Effectiveness of different methods of irrigation: conventional irrigation compared to active irrigation.
− Factors influencing irrigation: irrigant concentration, agitation time, taper of the root canal, temperature or depth of needle insertion needed.
− Efficacy of different methods of active irrigation systems.
− Different types of active irrigation: ultrasonic, sonic, laser or manual dynamic activation.
− Healing of apical periodontitis after using active irrigation or conventional type.

Amongst them, five of the articles were systematic reviews (two included a meta-analysis), one in vitro study, two in vivo studies, one ex vivo study, and one randomised controlled trial.

The quality assessment was performed by utilizing The Cochrane Handbook: Criteria for judging risk of bias. The risk-of-bias tool assesses the methodological quality of the studies by identifying biases and establishing their validity [33].

A table summarising the selected articles is shown below (Table 1).

## 3. Discussion

### 3.1. Effectiveness of Different Methods of Irrigation

There is a consensus in the endodontic society that the ultrasonic activation of the irrigant improves the canal’s cleanliness by eliminating the pulp tissue, dentine debris, and smear layer when compared to the conventional syringe. With irrigant activation techniques, the flow volume of the irrigant and the speed are higher when irrigating the canals, reaching more effective removal of debris, less apical blockage, and better access for the irrigant to the irregularities of the canals (non-instrumented areas and lateral canals). This is based on the use of each clinician without any standardized protocol of the IATs [11,13,14,19,36].

In addition, multisonic activation achieves better cleanliness than syringe irrigation and reduces bacteria and debris, like ultrasonic activation, especially on the apical third of the canal [16].

In a recent study, the authors quantified the extent of the air bubbles, known as vapour lock, which is a major problem when trying to disinfect by irrigating (these appear to happen more when the irrigant is delivered with the syringe needle). Kuah et al. [6] experimented on a small sample size (ten extracted teeth) to quantify the air bubbles and their number in the root canal system. The vapour lock occurred mainly in the apical third of the canal, the most complex and most complicated area to clean and disinfect, thus affecting the debridement and cleanliness. Despite the small sample size, this study sheds light on the importance of disinfecting the apical third and the use of IATs during root canal therapy. Further studies with larger samples, a standard irrigant, and clinical implications would be helpful in enhancing the success rate of root canal treatment [40].

This increased cleaning helps reduce the likelihood of persistent bacteria and prevents reinfection, thus reducing the possibility of treatment failure due to enhanced disinfection. These are clear advantages to using IATs when compared to CNI.

On the other hand, there is no direct correlation nor research between the use of IATs and the treatment outcome, healing of apical periodontitis in the long term, or statistically significant difference in effectiveness. The percentage of periapical healing is similar using activated or conventional irrigation. In addition, many variables are not taken into account in the studies, such as anatomy (apical delta, dentinal tubules), type of biofilm (community of microorganisms embedded in an extracellular matrix that adhere or attach to a surface), canal taper, power intensities of IATs, or the root filling itself; therefore, more studies are needed to understand the factors that influence the endodontic outcome [7,14,15,18,19,38].

### 3.2. Factors Influencing Irrigation

Irrigation is effective when there is an optimal chemo-mechanical dissolution or removal of the pulp tissue, the smear layer, and microorganisms from the root canal system. This depends on the duration, concentration of the irrigation procedure, and the flow of the irrigant, both being modifiable by irrigating activation systems. Higher concentrations of NaOCl cause it to penetrate more quickly, which causes the results to saturate at certain depths quickly. Therefore, the findings indicate that identical results can be obtained when lower quantities of NaOCl are provided over longer periods to promote tubular penetration. This continuous flow of irrigant and its penetration can be improved by only performing short vertical strokes (MDA) and not only using PUI or SI, showing superiority to CNI [11,38].

One of the CUI systems, Piezo Flow, was compared to EndoVac and Max-i-Probe, concluding that it was more relevant to the depth and volume of the irrigation rather than the technique [19].

In comparison to the equivalent coronal and middle segments, NaOCl infiltration was consistently lower apically. No single approach was able to achieve a greater effect over the whole root canal. SI exhibited the most coronal NaOCl penetration; in the middle third, all procedures performed similarly, with PUI and MDA outperforming SI [11]. According to this study, every technique would be appropriate for a certain area of the canal, and a combination of agitation methods might be required to obtain the maximum amount of irrigant penetration along the canal’s whole length. Additionally, compared to traditional needle irrigation, IATs greatly increase the tubular penetration of sodium hypochlorite throughout the root canal, particularly apically.

The diameter of the patency file (usually a manual K-file no.10 that goes 1 mm beyond the apical foramen) also influences the penetration of irrigants into the root canal. It allows the irrigant to penetrate the apical portion effectively and safely. For this to be effective, the irrigant will have enough concentration, time, and contact to dissolve the intracanal tissues. There is a risk of causing damage to the periapical tissues and extrusion of debris or irrigant if larger files are chosen [5].

Zeltner et al. [34] added that the irrigation solutions should be warmed, and their flow would increase the action of NaOCl. However, the authors concluded that the temperature and removing the necrotic pulp from accessory canals are independent.

### 3.3. Safety and Drawbacks

One of the risks of using NaOCl is its toxicity if it is extruded into periradicular tissues when irrigating. It is seen as iatrogenic harm to the patient [7,11].

This extrusion can happen more often in those teeth in which the apical constriction has been altered or eliminated during root canal preparation (perforations), a wide apical foramen (immature teeth), or resorption. This error impairs the standard of care overall and results in both intra- and post-operative pain. About 30% of teeth have extrusion, which is more common in teeth with resorption than in curved canals [15].

It may also occur more frequently while using a positive pressure system or manual irrigation. If the pressure inside the root canal is increased, there could be an apical extrusion of the irrigant and serious consequences, such as a “hypochlorite accident” [15,17]. If the equipment is handled with caution and training, accidents of this kind can be prevented.

### 3.4. Endovac

Endovac (which was described earlier) appears to be safer than manual, ultrasonic irrigation and other systems with fewer risks and consequences of extruding NaOCl in the periapex [15,35].

In addition, there are safety concerns when using ultrasonic energy for more than 2 min because the temperature is increased, and it is unknown how this could affect the bone and periodontal ligament [34].

#### IATs vs. CNI: Antimicrobial Efficacy and Intracanal Cleanliness

Suslia et al. [15] used 40 teeth for their study and compared the following methods using NaOCl as an irrigant: Endovac, EndoActivator, manual, and ultrasonic. There was no apparent difference between the methods used, the percentage of debris remaining, or if this determined the treatment outcome.

IATs are advised to be used throughout the root canal preparation process because, according to [14], they do cause greater debris and smear layer removal than CNI.

The NaOCl’s penetration into dentinal tubules is higher when using IATs as well as intracanal cleanliness [11,13,14,15,17,19,37].

Despite this, most of the available information on IATs originates from in vitro studies. It is still unknown how much of the microbial load is reduced when using active irrigation and if it is superior to the syringe method when treating real teeth [1]. Curved roots of molar teeth are needed in the studies due to their complex anatomy to better evaluate the effectiveness of agitation techniques [37]. Furthermore, the standardisation of research protocols is mentioned in all the selected articles in this review due to the high degree of heterogeneity that makes it nearly impossible to compare techniques, antimicrobial efficacy, or treatment outcomes. As a result, it is impossible to come to a consensus regarding which technique is better and more advantageous.

### 3.5. Call for Standardisation of Variables for Reliable Studies

#### 3.5.1. Radiopacity

A radiopaque material needs to be added to the irrigant solution (trying not to alter the properties of NaOCl) to study irrigant penetration with a radiographic technique [41].

Some authors [1,39] outlined how several physical “properties such as the solution’s surface tension and its contact angle on dentine” can react differently, meaning that radiopaque solutions are not dependable irrigant alternatives.

#### 3.5.2. Type of Sample

It is preferable to use the intact pulp in extracted teeth instead of transparent acrylic blocks or 3D-printed teeth with artificially placed tissue because the different materials and surfaces can affect irrigant penetration (real teeth have hydrophilic dentine, and resins are hydrophobic, which may favour the entrapment of air bubbles), although this is not very well understood nor its interaction with the biofilm, pulp tissue, or dentine debris [39].

#### 3.5.3. Irrigant Delivery and Its Activation

There is a need to acknowledge the needle type, size, or insertion depth used in the studies so that they can be compared to the current clinical standards. Some studies do not mention the volume or its flow, and others use large needles (>27 G) for syringe irrigation, which deviates from the current standards. The larger the number of the gauge, the smaller and thinner the needle will be. A 27 G (0.42 mm of external diameter) or 30 G (0.32 mm of diameter) needle is advised in the endodontic field [1].

Vera et al. in 2012 [41] explained the need to standardise the application time, volume, and activation method for better and higher-quality clinical studies.

To examine the in vivo effects of IATs on clinical outcomes and periapical healing after treatment, it is necessary to standardise experimental protocols and create a more representative research model because the available data are too inconsistent to compare and determine the superiority of a single technique [14].

Vera et al. [5] also point out that the power settings of the ultrasound devices should be identical. Furthermore, they found in their study that this was not the case and that various manufacturers created the ultrasound equipment, or that certain information was left out of the studies that were examined. The authors also state that while employing ultrasonic activation, the number of activation cycles should be comparable, in addition to keeping the overall activation period consistent.

#### 3.5.4. Healing of Apical Periodontitis

There is only one randomised controlled clinical trial [38] that provides information on the primary root canal treatment outcome. This study did not find a remarkable difference between syringe irrigation and ultrasonic activation groups, although the ultrasonic one was favoured for the irrigation protocol. According to Căpută et al. [1], almost all studies they compared, clinical and in vitro, concluded that ultrasonic activation was superior to syringe irrigation (more debris and pulp remnants removed from the root canal system). Still, only half of the in vitro studies found a superior antimicrobial effect.

## 4. Conclusions

Active irrigation systems appear to be more effective than conventional syringes when performing a root canal treatment. The irrigation is improved along with its penetration in complex areas, and it removes more debris and pulpal remnants as well as killing more bacteria inside the root canal irregularities.

On the other hand, there is no clear evidence that active irrigation influences the treatment outcome or the healing of apical periodontitis. This is not well understood due to a lack of research on the antimicrobial efficacy and treatment outcomes while employing ultrasonic irrigation or any IAT method.

Furthermore, current endodontic studies and articles lack high-quality data and standardized protocols to be able to compare the effectiveness of IATs on antimicrobial efficacy, periapical healing or treatment outcome. A unique, standardized clinical procedure cannot be achieved due to the great diversity in these and the methods utilized (various sample sizes, types of teeth or blocks, irrigation activation systems, volumes, or times, among others). The dental community would benefit from having a standard protocol for the use of IATs in clinical practice. This should be pursued through further investigation in the field.

Active irrigation has been present for decades and has been gaining more popularity due to its effectiveness. Ultrasonic, sonic, negative pressure systems, or multisonic activation devices are commonly selected in clinical practice, but because of their high costs, the conventional syringe is still the most used method.

To determine the optimal irrigating technique, it may be concluded that further standardized research is required, with an emphasis on antibacterial efficacy, the healing of apical periodontitis, and treatment outcomes.

## Figures and Tables

**Table 1:** Summary of the selected articles.

Title	Authors	Objectives	Conclusions
Temperature Changes During Ultrasonic Irrigation with Different Inserts and Modes of Activation	[34]	To measure intracanal temperatures while using different methods of active irrigation.	PUI after syringe use is associated with a rise in the temperatures of the irrigant. More research is needed to observe if ultrasonic activation is responsible for a higher irrigation efficacy
Efficacy of Different Irrigation and Activation Systems on the Penetration of Sodium Hypochlorite into Simulated Lateral Canals and Up to Working Length: An In Vitro Study	[13]	To assess how effectively the irrigation and activation systems currently in use affect NaOCl’s penetration into simulated lateral canals and up to working length in a closed system.	The root canal system was effectively irrigated up to working length by ANP irrigation. In contrast, ANP showed just a small amount of irrigant activation in non-instrumented regions, evident in lateral canals. The PUI group showed much greater irrigant penetration into lateral canals.
Comparison of the Debridement Efficacy of the EndoVac Irrigation System and Conventional Needle Root Canal Irrigation In Vivo	[35]	To compare the debridement efficacy of conventional needle irrigation versus EndoVac system in vivo.	EndoVac irrigation had less debris at 1 mm from WL compared with conventional needle irrigation. However, there was no significant difference at the 3-mm level.
Disinfection of Root Canals with Photon-initiated Photoacoustic Streaming	[36]	To compare the efficacy of laser-activated and ultrasonically activated root canal disinfection with conventional irrigation.	Activated disinfection did not completely remove bacteria from the apical third and infected dentinal tubules. Laser activation left less apical bacteria when compared to ultrasonic activation.
Activated Irrigation vs. Conventional non-activated Irrigation in Endodontics—A Systematic Review	[15]	To determine the clinical efficiency of activated irrigants and conventional irrigation.	Irrigant activation appears to be beneficial for postoperative pain intensity, and debridement of canal and isthmus. No evidence regarding antibacterial effect or long-term healing.
Effect of Maintaining Apical Patency on Irrigant Penetration into the Apical Third of Root Canals When Using Passive Ultrasonic Irrigation: An In Vivo Study	[5]	To ascertain if the application of a patency file, following passive ultrasonic activation, is associated with the presence of radiopaque irrigating solution in the apical third of root canals.	Using PUI improves the delivery of irrigant into the apical third of root canals while maintaining apical patency.
Review of ultrasonic irrigation in endodontics: increasing action of irrigating solutions	[19]	To summarise and discuss the available information regarding ultrasonic irrigation in endodontics.	Ultrasonic irrigation improved cleanliness in the canal and removal of smear layer and bacteria. However, there is a need to standardise protocols and be able to correlate the clinical efficacy of ultrasonic devices with improved treatment outcomes.
Root canal debridement efficacy of different final irrigation protocols	[37]	To assess the extent to which four root canal irrigation techniques removed debris and the smear layer, as well as how well they removed any remaining soft tissues in curved root canals.	CanalBrush removed dirt and the smear layer just as well as passive ultrasonic irrigation. To assess the efficacy of this regimen considering irrigant volume variations and the impact of root canal system configuration, more research is necessary.
Radiographic Healing after a Root Canal Treatment Performed in Single-rooted Teeth with and without Ultrasonic Activation of the Irrigant: A Randomized Controlled Trial	[38]	To evaluate the results of a root canal procedure both with and without additional ultrasonic irrigant activation.	Equal contributions to periapical healing with and without ultrasonic stimulation of the irrigant.
Efficacy of irrigant activation techniques in removing intracanal smear layer and debris from mature permanent teeth: a systematic review and meta-analysis	[14]	To determine if irrigant activation techniques (IATs) induce more intracanal smear layer and debris removal compared to conventional needle irrigation (CNI).	Using IATs during root canal preparation is advised since they enhance intracanal cleanliness. Standardising experimental procedures and creating a more representative research model is necessary to examine how in vivo studies impact clinical results and periapical healing after receiving root canal therapy.
Ultrasonic Irrigant Activation during Root Canal Treatment: A Systematic Review	[1]	To examine the data about the healing of apical periodontitis and the cleaning and disinfection of root canals when ultrasonic irrigant activation is used during primary root canal treatment of mature permanent teeth as opposed to syringe irrigation.	Since there was insufficient evidence to make firm clinical recommendations, none could be made. Future research should focus on the antibacterial activity effect and healing of apical periodontitis in teeth with multiple root canals.
The influence of irrigant activation, concentration and contact time on sodium hypochlorite penetration into root dentine: an ex vivo experiment.	[11]	To determine whether, in comparison to conventional needle irrigation (CNI), irrigant activation techniques such as manual dynamic activation (MDA), passive ultrasonic irrigation (PUI), and sonic irrigation (SI) improve tubular penetration of sodium hypochlorite (NaOCl) into root dentine. Second, examine if these methods work better when the concentration of NaOCl is increased or the contact time is extended.	NaOCl penetration into root dentine was enhanced by agitating irrigants with MDA, PUI, or SI, as well as by utilising higher irrigant concentrations or contact times. Longer exposure times to NaOCl at lower concentrations, however, led to tubular penetration depths that were comparable to those at higher concentrations.
A critical analysis of research methods and experimental models to study irrigants and irrigation systems	[39]	Evaluate experimental techniques and models used for examining irrigants and irrigation systems critically to suggest future research options.	Research techniques should be evolving. Additionally, models must be verified. There are no perfect approaches or models that always yield all the answers and function flawlessly under all circumstances.
Irrigating Solutions and Activation Methods Used in Clinical Endodontics: A Systematic Review	[7]	To assess the limitations of irrigating solutions and clinical endodontic procedures for disinfecting the root canal system in patients with apical periodontitis.	High heterogeneity in the available data to compare and determine which useful irrigation strategies are best in each therapeutic scenario. Standardising application times, volumes, and activation techniques will help identify the best irrigating techniques to lower the bacterial load and increase the predictability of endodontic therapy.
Micro-CT Evaluation of Different Root Canal Irrigation Protocols on the Removal of Accumulated Hard Tissue Debris: A Systematic Review and Meta-Analysis	[17]	To carry out a meta-analysis to assess the degree to which several irrigation methods remove AHTD, which would aid in the creation of an evidence-based root canal irrigation protocol.	When it comes to debris removal, PUI is better than SNI; nevertheless, AHTD removal performance is greater with SWEEPS and PIPS. This finding implies that PIPS and SWEEPS would be a superior choice for difficult-to-clean root canals. In contrast, the negative pressure system and mechanical-activated system were shown to be less effective
Effect of Chemical Debridement and Irrigant Activation on Endodontic Treatment Outcomes: An Updated Overview	[16]	To provide an overview of the impact of different irrigant activation and agitation methods and tools on the outcome of endodontic treatment	Future research should pay closer attention to each technique’s antibacterial activity and how it affects the healing of apical periodontitis. Recent developments like multisonic and laser activation are also interesting techniques that require additional clinical studies to demonstrate their efficacy.

## Data Availability

Data can be made available by asking the corresponding author.
